# The Association Between Social Determinants of Health and HIV Risk Behaviors and HIV Testing Among Sexual and Gender Minority Individuals: A National Survey Study

**DOI:** 10.1007/s10461-025-04895-5

**Published:** 2025-11-12

**Authors:** Edith Nnenna Utaka, Xueying Yang, Fanghui Shi, Huiyi Xia, Qingyang Li, Brooks Yelton, Daniela Friedman, Lorie Donelle, Xiaoming Li

**Affiliations:** 1 Department of Health Promotion, Education and Behavior, Arnold School of Public Health, University of South Carolina, Columbia, SC 29208 USA; 2 South Carolina SmartState Center for Healthcare Quality, Arnold School of Public Health, University of South Carolina, Columbia, USA SC 29208; 3 College of Nursing, University of South Carolina, Columbia, SC 29208 USA

**Keywords:** Sexual and gender minority, HIV testing, HIV risk behavior, Social determinants of health

## Abstract

Social determinants of health (SDOH) predispose Sexual and Gender Minority (SGM) individuals to HIV infection through risky behaviors and less HIV testing. This study used data from the 2022 Behavioral Risk Factor Surveillance System (BRFSS) survey to evaluate the association between composite measures of SDOH and HIV risk behaviors and HIV testing among adult SGM population in the US. SGM individuals were identified through two questions that asked about respondents’ sexual orientation and gender identity. Two primary outcomes of interest were HIV testing behavior and HIV risk behavior. SDOH was measured by ten items which were summed to get a composite score, where a higher score indicates a worse social disadvantage. Logistic regression models were used to examine the association of SDOH composite score with HIV risk behaviors and HIV testing. Among 10,774 SGM individuals who provided responses to the HIV testing question, 5,696 (52.87%) reported lifetime HIV testing. For the HIV risk behavior question, 10,736 SGM individuals responded, of whom 2,059 (19.18%) reported engaging in HIV risk behaviors. SGMs with more social disadvantages were more likely to engage in HIV risk behaviors (Adjusted Odds ratio (AOR) = 1.15, 95% Confidence interval (CI) = 1.12, 1.19) and have a high likelihood of HIV testing (AOR = 1.09, 95% CI = 1.06, 1.12) compared with those with less social disadvantages. A high composite SDOH score is associated with increased HIV risk behaviors and high HIV testing among SGM individuals. Reducing the HIV burden among the SGM population requires addressing structural factors through multifaceted approaches.

**Supplementary Information:**

The online version contains supplementary material available at 10.1007/s10461-025-04895-5.

## Introduction

Sexual and Gender Minority (SGM) individuals are people whose sexual orientation is not exclusively heterosexual, or whose gender identity differs from the sex assigned to them at birth. They include but are not limited to people who identify as lesbian, gay, bisexual, asexual, transgender, non-binary, Two-Spirit, queer, or intersex [1]. According to a systematic review and meta-analysis by Kloek et al. in 2022, 67% of new HIV infections were among gay, bisexual, and other men who reported having male-to-male sexual contact [2]. Another systematic review recorded a global HIV prevalence of 19.9% among trans females and 2.56% among trans males [3]. Increased incidence of HIV among SGM individuals has been associated with a high prevalence of HIV risky behaviors. In a meta-analysis among adolescent sexual minority males in the United States (US), 67% of the respondents had sex with any gender within 3–6 months before the study, 50% reported no condom use at last sex, 32% reported the use of alcohol or drugs in their last sex, while 44% had condomless anal sex within 6 months before the study [4].

In the US, HIV testing among SGM groups varies, with studies reporting 26.6% to 68% of the population being tested [5, 6]. The Centers for Disease Control and Prevention (CDC) recommends at least one annual HIV screening for people at risk of HIV; however, in 2017 and 2023, only 77% and 78% of gay and bisexual men in the US screened for HIV, respectively [7]. This falls short of the recommended 100% annual screening among this population, thus highlighting the need to promote more testing among this population. Poor adherence to routine HIV screening leads to undiagnosed HIV infections or late diagnosis of HIV among SGM.

While risky sexual behaviors and low HIV testing rates increase the risk of HIV infection among SGM individuals, social determinants of health (SDOH) have been shown to influence both behaviors. SDOH are the conditions in which people are born, grow, work, live, and age, and the wider set of forces and systems shaping the conditions of daily life [8]. Transgender people and men who have sex with men (MSM) face marginalization, violence, stigma, discrimination, social rejection, and exclusion, which impedes their access to primary HIV prevention and treatment services, impacts their overall health and well-being, and increases their likelihood of acquiring HIV infection [9–11].

Past studies have demonstrated associations between individual SDOH, like education, material hardship, income, and housing quality, with HIV risky behaviors and HIV testing. African American MSM were more likely to engage in transactional sex if they did not complete high school, and this association was also observed if their neighbors did not complete high school [12]. Adolescent sexual minority males who have sex with males, and who experience material hardship, have multiple male sexual partners with higher odds of engaging in condomless anal intercourse with their male partners [13]. Among MSM in Los Angeles, social capital resources were associated with a lower probability of methamphetamine use during sex and a higher probability of HIV testing, while experiences of homophobia were associated with a higher probability of methamphetamine use during sex, giving or receiving money or drugs for sex, and missed appointments with HIV care providers [14]. Studies among Hispanic/Latino gay, bisexual, and other MSM showed that HIV testing is positively associated with higher levels of education, higher income, access to healthcare services, insurance, disclosure of sexual identity to a healthcare provider, and social support [15–17].

Experiences with SDOH are interrelated and interdependent, and their influence on health is due to an overall burden rather than an individual experience [18]. The clustering of SDOH within individuals suggests that the cumulative burden of SDOH could be a meaningful predictor of health outcomes than a single determinant considered in isolation [19]. Exploring the complexity of coexisting SDOH may provide insight into the effect of the accumulation of social influences on health [19]. In addition, a composite score can help identify individuals at higher cumulative risk, guiding more targeted public health interventions to address multiple social barriers simultaneously. Although composite measures of SDOH have been used to explore associations with some health outcomes, such as general health status and prevalence of arthritis [20, 21], no study has examined the combined influence of SDOH variables on HIV risk behaviors and HIV testing among SGM individuals in the US.

This study aimed to evaluate the association between composite measures of some SDOH and HIV risk behaviors and HIV testing among the SGM population in the United States using nationwide population data from the 2022 Behavioral Risk Factor Surveillance System (BRFSS) survey.

## Methods

### Data Source and Cohort Definition

In 2013, the CDC developed a module for the BRFSS to collect data on sexual orientation and gender identity, thus providing generalizable data for the SGM population [22]. This study utilized publicly available secondary data collected in 2022 from the BRFSS across 39 states, the District of Columbia, and two territories (Puerto Rico and the US Virgin Islands). BRFSS is a system of ongoing annual cross-sectional telephone surveys conducted by the CDC in collaboration with U.S. states and territories. The survey collects data on health-related risk behaviors, chronic health conditions, healthcare access, and use of preventive services from the noninstitutionalized adult population (≥ 18 years) residing in the United States and participating areas [23].

### Sampling Methods

This is a cross-sectional study using data from BRFSS, which utilizes a complex, probability-based sampling design to obtain data representative of non-institutionalized adults in the US. The survey uses a dual-frame sampling method consisting of random-digit-dialing of both landline and cellular telephone numbers. Within households contacted by landline, one adult is randomly selected, while eligible adults answering cellular calls are invited to participate. BRFSS ensures representativeness of the sample by applying the raking weighting procedures to the collected data.

### Collected Data

The BRFSS questionnaire consists of three components: (1) the core set of questions administered by all participating states that covers demographics, health behaviors, chronic conditions and preventive health practices; (2) the optional modules which allows states to choose from, and covers specific topics such as sexual health; (3) state-added questions which allows states to add their own questions to address state-specific public health issues.

In this study, we utilized variables obtained from the BRFSS core questionnaire and the optional social determinants of health and Health Equity module (SDOH/HE). The variables extracted for our study include demographic information such as race, age, sex at birth, level of education, sexual identity and gender orientation, history of chronic diseases, HIV testing behavior, and HIV risk behaviors. The SDOH variables extracted include housing stability and quality, food security, transportation access, utility security, loneliness, social and emotional support, life satisfaction, and mental well-being.

### Study Population

Our study population is SGM individuals who were identified through two questions that asked about the respondent’s sexual orientation and gender identity. The question about sexual orientation was “Which of the following best represents how you think of yourself?” with response categories of “Lesbian or Gay”, “Straight, that is not gay or lesbian”, “Bisexual, “Something else”, “I don’t know the answer”, and “Refused”. Respondents who selected “Lesbian or Gay” or “Bisexual” were classified as sexual minorities (SM). The question about sexual identity was “Do you consider yourself to be transgender?” with response categories of “Yes, Transgender, male-to-female”, “Yes, Transgender, female-to-male”, “Yes, Transgender, gender nonconforming”, “No”, “Don’t know/not sure”, and “Refused”. Respondents who answered “Yes” to any transgender identity were classified as Gender minorities (GM).

Based on these responses, the overall SGM study population includes individuals who identified as SM, GM, or both. We further categorized SGM participants into three mutually exclusive groups: [1] sexual minority-only group (SM only) including individuals identifying as gay, bisexual, lesbian, or other non-heterosexual orientation but not gender minority; [2] gender minority-only group (GM only) comprising male-to-female transgenders, female-to-male transgenders, and gender nonconforming individuals but not sexual minorities; and [3] sexual and gender minority group (SM & GM) consisting of individuals who were identified as both gender minority and sexual minority.

The inclusion criteria for our study are any self-identified SGM population who responded to questions about either HIV testing or HIV risk behavior. Based on these criteria, the sample size for our HIV testing behavior outcome is 10,774, while the sample size for the HIV risk behavior outcome is 10,736. Weighting by the ranking process was applied to the BRFSS data set, making it representative of the adult population.

### Outcomes

#### HIV Risk Behavior

One question was used to assess HIV risk behaviors. Respondents were asked if they had either injected any drug other than those prescribed for them in the past year, been treated for a sexually transmitted disease or STD in the past year, given or received money or drugs in exchange for sex in the past year, had anal sex without a condom in the past year, had four or more sex partners in the past year. The response options to this question include “Yes”, “No”, “Refused to answer”, and “Unknown/Unsure”.

#### HIV Testing Definition

For HIV testing, individuals were asked if they had ever been tested for HIV, including fluid testing from their mouths, but not including tests they may have had for blood donation. The response options to this question include “Yes”, “No”, “Refused to answer”, and “Unknown/Unsure”.

### SDOH

SDOH data were obtained from questions in the SDOH/HE module and included ten questions about employment/economic stability, housing security and quality, food security, transportation access, utility security, loneliness, social and emotional support, life satisfaction, and mental well-being. The respondents were asked questions about their experiences with different SDOH variables. The response options for the various questions vary, and detailed information about the specific SDOH questions and their response options can be found at the BRFSS website(https://www.cdc.gov/brfss/questionnaires/pdf-ques/2022-BRFSS-Questionnaire-508.pdf).

Responses to the individual SDOH variables were recoded to a value of 0 or 1, with 0 indicating a lower degree of SDOH disadvantage and 1 indicating a higher degree of SDOH disadvantage. The responses of “don’t know/not sure,” “refused to answer”, or “missing responses” to any of the questions were recoded as “missing” (see Supplementary Table 3 for recoding of the SDOH variables). The SDOH composite score was calculated by summing up each score of the ten individual SDOH item variables, yielding a total score ranging from 0 to 10. Higher scores indicate greater social disadvantage. The internal consistency of the scale was assessed, with a Cronbach’s alpha of 0.723 (95% CI: 0.721–0.726).

### Sociodemographic

Sociodemographic information was obtained and includes information about race (e.g., Non-Hispanic White, Non-Hispanic Black, age (e.g., 18–34, 35–54), BMI (e.g., Normal Weight, Underweight), sex (male and female), SGM (SM only, GM only, and SM &GM), educational level (e.g., < High School, >=High School/Graduate), employment status (e.g., Employed, Unemployed, etc.), income level (e.g., < 25,000, 25,000–50,000), health insurance (e.g., yes, no), and affordability seeing doctors (e.g., yes, no).

### Data Analysis

Data analysis was done using R statistical software. Descriptive statistics were used to describe frequencies, percentages, and means, which were presented as tables. A logistic regression model was developed to examine the association of the SDOH composite score with HIV risk behaviors and HIV testing, adjusting for some other social demographic characteristics as confounders. We also conducted a logistic regression analysis to understand the association between individual SDOH and HIV risk behaviors and HIV testing. All analyses were based on a 5% level of significance with a 95% confidence interval. The descriptive statistic included all the SGM respondents who responded to either the HIV testing (n=10,774) or HIV risk behavior questions (n=10,736). In the logistic regression models, we excluded participants with any missing data either on the outcome measures (HIV testing or HIV risk behaviors) or the SDOH items given the minimal level of missingness (a maximum of 5% across all variables), yielding a total sample size of 9,819 (94.4%) for HIV test analysis and 10,111 (94.6%) for HIV risk behavior analysis. 

## Results

### Sociodemographic Characteristics

Table 1 illustrates the distribution of sociodemographic variables and SDOH for our respondents by HIV testing and HIV risk behaviors. Out of the 10,774 SGM individuals that responded to the HIV testing question, 5,696 (52.87%) have been tested in their lifetime, 4705 (43.67%) have never been tested, 13 (0.12%) refused to provide an answer, while 360 (3.34%) responded unknown/unsure. For HIV risk behavior, out of the 10,736 SGM individuals who responded, 2059 (19.18%) engaged in risky behaviors, 8,626 (80.35%) did not engage in risky behavior, 39 (0.36%) refused to provide an answer, while 12 (0.11%) responded unknown/unsure.

**Table 1:** Sociodemographic distribution and social determinants of health by HIV testing status (*N* = 10774) and HIV risk behaviors (*N* = 10736)

Factors	Total (*N* = 10774)	HIV Testing		Total (*N* = 10736)	HIV Risk Behavior	
		Yes (*N* = 5696) 52.87%	No (*N* = 4705) 43.67%		Yes (*N* = 2059) 19.18%	No (*N* = 8626) 80.35%
Race						
Non-Hispanic White	8056 (74.8%)	4152 (72.9%)	3591 (76.3%)	8030 (74.8%)	1433 (69.6%)	6558 (76.0%)
Non-Hispanic Black	637 (5.9%)	444 (7.8%)	185 (3.9%)	633 (5.9%)	139 (6.8%)	493 (5.7%)
Hispanic	1144 (10.6%)	615 (10.8%)	503 (10.7%)	1139 (10.6%)	280 (13.6%)	852 (9.9%)
Other	937 (8.7%)	485 (8.5%)	426 (9.1%)	934 (8.7%)	207 (10.1%)	723 (8.4%)
Age						
18–34	4764 (44.2%)	2176 (38.2%)	2427 (51.6%)	4743 (44.2%)	1204 (58.5%)	3514 (40.7%)
35–54	3003 (27.9%)	2057 (36.1%)	844 (17.9%)	2995 (27.9%)	596 (28.9%)	2385 (27.6%)
55–64	1299 (12.1%)	761 (13.4%)	504 (10.7%)	1293 (12.0%)	157 (7.6%)	1131 (13.1%)
65+	1623 (15.1%)	663 (11.6%)	889 (18.9%)	1620 (15.1%)	93 (4.5%)	1524 (17.7%)
Unknown/Refused	85 (0.8%)	39 (0.7%)	41 (0.9%)	85 (0.8%)	9 (0.4%)	72 (0.8%)
BMI						
Normal Weight	3136 (29.1%)	1619 (28.4%)	1412 (30.0%)	3120 (29.1%)	646 (31.4%)	2459 (28.5%)
Underweight	247 (2.3%)	112 (2.0%)	129 (2.7%)	246 (2.3%)	55 (2.7%)	189 (2.2%)
Overweight	6830 (63.4%)	3722 (65.3%)	2881 (61.2%)	6811 (63.4%)	1278 (62.1%)	5510 (63.9%)
Unknown/Refused	561 (5.2%)	243 (4.3%)	283 (6.0%)	559 (5.2%)	80 (3.9%)	468 (5.4%)
Sex						
Male	4485 (41.6%)	2658 (46.7%)	1727 (36.7%)	4473 (41.7%)	1228 (59.6%)	3216 (37.3%)
Female	6289 (58.4%)	3038 (53.3%)	2978 (63.3%)	6263 (58.3%)	831 (40.4%)	5410 (62.7%)
SGM						
SM only	9557 (88.7%)	5188 (91.1%)	4039 (85.8%)	9520 (88.7%)	1847 (89.7%)	7629 (88.4%)
GM only	729 (6.8%)	275 (4.8%)	425 (9.0%)	728 (6.8%)	96 (4.7%)	629 (7.3%)
SM & GM	488 (4.5%)	233 (4.1%)	241 (5.1%)	488 (4.5%)	116 (5.6%)	368 (4.3%)
Education Level						
< High School	465 (4.3%)	219 (3.8%)	232 (4.9%)	464 (4.3%)	94 (4.6%)	367 (4.3%)
>=High School/Graduate	10,287 (95.5%)	5472 (96.1%)	4460 (94.8%)	10,250 (95.5%)	1960 (95.2%)	8243 (95.6%)
Unknown	22 (0.2%)	5 (0.1%)	13 (0.3%)	22 (0.2%)	5 (0.2%)	16 (0.2%)
Employment Status						
Employed	6717 (62.3%)	3787 (66.5%)	2706 (57.5%)	6691 (62.3%)	1453 (70.6%)	5208 (60.4%)
Unemployed	1483 (13.8%)	628 (11.0%)	806 (17.1%)	1478 (13.8%)	328 (15.9%)	1140 (13.2%)
Retired/Homemaker	1776 (16.5%)	778 (13.7%)	924 (19.6%)	1771 (16.5%)	144 (7.0%)	1624 (18.8%)
Unable to work	709 (6.6%)	460 (8.1%)	228 (4.8%)	708 (6.6%)	114 (5.5%)	592 (6.9%)
Unknown/Refused	89 (0.8%)	43 (0.8%)	41 (0.9%)	88 (0.8%)	20 (1.0%)	62 (0.7%)
Income Level						
< 25,000	1506 (14.0%)	848 (14.9%)	612 (13.0%)	1498 (14.0%)	314 (15.3%)	1178 (13.7%)
25,000–50,000	2430 (22.6%)	1308 (23.0%)	1054 (22.4%)	2417 (22.5%)	480 (23.3%)	1929 (22.4%)
50,000–200,000	4571 (42.4%)	2541 (44.6%)	1880 (40.0%)	4559 (42.5%)	851 (41.3%)	3697 (42.9%)
> 200,000	546 (5.1%)	341 (6.0%)	189 (4.0%)	544 (5.1%)	137 (6.7%)	405 (4.7%)
Unknown	1721 (16.0%)	658 (11.6%)	970 (20.6%)	1718 (16.0%)	277 (13.5%)	1417 (16.4%)
Health Insurance						
Yes	9672 (89.8%)	5229 (91.8%)	4106 (87.3%)	9641 (89.8%)	1804 (87.6%)	7796 (90.4%)
No	657 (6.1%)	322 (5.7%)	325 (6.9%)	651 (6.1%)	180 (8.7%)	466 (5.4%)
Unsure/Unknown	445 (4.1%)	145 (2.5%)	274 (5.8%)	444 (4.1%)	75 (3.6%)	364 (4.2%)
Personal Health Care Provider						
Yes	8615 (82.8%)	3781 (80.4%)	4834 (84.9%)	8898 (82.9%)	1652 (80.2%)	7206 (83.5%)
No	1707 (16.4%)	882 (18.7%)	825 (14.5%)	1754 (16.3%)	391 (19.0%)	1353 (15.7%)
Unknown	79 (0.8%)	42 (0.9%)	37 (0.6%)	84 (0.8%)	16 (0.8%)	67 (0.8%)
Affordability Seeing Doctors						
Yes	1705 (15.8%)	968 (17.0%)	679 (14.4%)	1690 (15.7%)	425 (20.6%)	1257 (14.6%)
No	9033 (83.8%)	4714 (82.8%)	4005 (85.1%)	9010 (83.9%)	1629 (79.1%)	7340 (85.1%)
Unknown	36 (0.3%)	14 (0.2%)	21 (0.4%)	36 (0.3%)	5 (0.2%)	29 (0.3%)
Asthma History						
No	8227 (76.4%)	4222 (74.1%)	3727 (79.2%)	8198 (76.4%)	1554 (75.5%)	6607 (76.6%)
Yes	2492 (23.1%)	1446 (25.4%)	952 (20.2%)	2484 (23.1%)	494 (24.0%)	1977 (22.9%)
Unknown	55 (0.5%)	28 (0.5%)	26 (0.6%)	54 (0.5%)	11 (0.5%)	42 (0.5%)
COPD History						
No	9969 (92.5%)	5176 (90.9%)	4452 (94.6%)	9936 (92.5%)	1928 (93.6%)	7959 (92.3%)
Yes	764 (7.1%)	496 (8.7%)	238 (5.1%)	760 (7.1%)	125 (6.1%)	633 (7.3%)
Unknown	41 (0.4%)	24 (0.4%)	15 (0.3%)	40 (0.4%)	6 (0.3%)	34 (0.4%)
Social Determinants of Health (SDOH)						
Life Satisfaction						
Satisfied/Very satisfied	9224 (85.6%)	4860 (85.3%)	4054 (86.2%)	9224 (85.9%)	1675 (81.4%)	7517 (87.1%)
Dissatisfied/Very dissatisfied	1283 (11.9%)	699 (12.3%)	540 (11.5%)	1283 (12.0%)	345 (16.8%)	932 (10.8%)
Missing	267 (2.5%)	137 (2.4%)	111 (2.4%)	229 (2.1%)	39 (1.9%)	177 (2.1%)
Getting Social and Emotional Support						
Always/Usually	7408 (68.8%)	3903 (68.5%)	3263 (69.4%)	7408 (69.0%)	1313 (63.8%)	6067 (70.3%)
Sometimes	2109 (19.6%)	1127 (19.8%)	905 (19.2%)	2109 (19.6%)	464 (22.5%)	1638 (19.0%)
Rarely/Never	1024 (9.5%)	552 (9.7%)	438 (9.3%)	1024 (9.5%)	255 (12.4%)	765 (8.9%)
Missing	233 (2.2%)	114 (2.0%)	99 (2.1%)	195 (1.8%)	27 (1.3%)	156 (1.8%)
Feeling Social Isolation						
Rarely/Never	5483 (50.9%)	2862 (50.2%)	2443 (51.9%)	5483 (51.1%)	940 (45.7%)	4522 (52.4%)
Sometimes	3427 (31.8%)	1800 (31.6%)	1502 (31.9%)	3427 (31.9%)	706 (34.3%)	2711 (31.4%)
Always/Usually	1639 (15.2%)	911 (16.0%)	672 (14.3%)	1639 (15.3%)	382 (18.6%)	1248 (14.5%)
Missing	225 (2.1%)	123 (2.2%)	88 (1.9%)	187 (1.7%)	31 (1.5%)	145 (1.7%)
Lost or Reduced Employment						
No	8767 (81.4%)	4553 (79.9%)	3919 (83.3%)	8767 (81.7%)	1537 (74.6%)	7198 (83.4%)
Yes	1769 (16.4%)	1022 (17.9%)	684 (14.5%)	1769 (16.5%)	487 (23.7%)	1275 (14.8%)
Missing	238 (2.2%)	121 (2.1%)	102 (2.2%)	200 (1.9%)	35 (1.7%)	153 (1.8%)
Receiving Food Stamps						
No	9102 (84.5%)	4667 (81.9%)	4125 (87.7%)	9102 (84.8%)	1694 (82.3%)	7373 (85.5%)
Yes	1456 (13.5%)	920 (16.2%)	486 (10.3%)	1456 (13.6%)	330 (16.0%)	1124 (13.0%)
Missing	216 (2.0%)	109 (1.9%)	94 (2.0%)	178 (1.7%)	35 (1.7%)	129 (1.5%)
Food Not Lasting/No Money to Buy						
Rarely/Never	8921 (82.8%)	4644 (81.5%)	3975 (84.5%)	8921 (83.1%)	1603 (77.9%)	7286 (84.5%)
Sometimes	1039 (9.6%)	583 (10.2%)	418 (8.9%)	1039 (9.7%)	259 (12.6%)	775 (9.0%)
Always/Usually	593 (5.5%)	354 (6.2%)	219 (4.7%)	593 (5.5%)	158 (7.7%)	430 (5.0%)
Missing	221 (2.1%)	115 (2.0%)	93 (2.0%)	183 (1.7%)	39 (1.9%)	135 (1.6%)
Unable to Pay Mortgage/Rent/Utility						
No	8899 (82.6%)	4545 (79.8%)	4045 (86.0%)	8899 (82.9%)	1576 (76.5%)	7290 (84.5%)
Yes	1613 (15.0%)	1023 (18.0%)	545 (11.6%)	1613 (15.0%)	438 (21.3%)	1168 (13.5%)
Missing	262 (2.4%)	128 (2.2%)	115 (2.4%)	224 (2.1%)	45 (2.2%)	168 (1.9%)
Threatened to Shut Off Utility Services						
No	9507 (88.2%)	4897 (86.0%)	4276 (90.9%)	9507 (88.6%)	1743 (84.7%)	7730 (89.6%)
Yes	1012 (9.4%)	664 (11.7%)	325 (6.9%)	1012 (9.4%)	266 (12.9%)	740 (8.6%)
Missing	255 (2.4%)	135 (2.4%)	104 (2.2%)	217 (2.0%)	50 (2.4%)	156 (1.8%)
Lack of Reliable Transportation						
No	9160 (85.0%)	4774 (83.8%)	4079 (86.7%)	9160 (85.3%)	1646 (79.9%)	7480 (86.7%)
Yes	1364 (12.7%)	793 (13.9%)	519 (11.0%)	1364 (12.7%)	365 (17.7%)	992 (11.5%)
Missing	250 (2.3%)	129 (2.3%)	107 (2.3%)	212 (2.0%)	48 (2.3%)	154 (1.8%)
Feeling Stress Within the Last 30 Days						
Rarely/Never	4725 (43.9%)	2398 (42.1%)	2174 (46.2%)	4708 (44.1%)	3984 (46.2%)	724 (35.2%)
Sometimes	3095 (28.7%)	1644 (28.9%)	1321 (28.1%)	3086 (28.9%)	2447 (28.4%)	639 (31.0%)
Always/Usually	2687 (24.9%)	1513 (26.6%)	1097 (23.3%)	2675 (25.0%)	2030 (23.5%)	645 (31.3%)
Missing	267 (2.5%)	141 (2.5%)	113 (2.4%)	216 (2.0%)	165 (1.9%)	51 (2.5%)
SDOH Score						
Mean (SD)	1.96 (2.09)	2.12 (2.21)	1.78 (1.92)	1.96 (2.09)	2.50 (2.35)	1.84 (1.99)
Median [Min, Max]	1.00 [0, 10.0]	1.00 [0, 10.0]	1.00 [0, 10.0]	1.00 [0, 10.0]	2.00 [0, 10.0]	1.00 [0, 10.0]
Missing	632 (5.9%)	301 (5.3%)	281 (6.0%)	594 (5.5%)	105 (5.1%)	469 (5.4%)
HIV Prevalence Rate (per 100,000 population)						
Mean (SD)	303 (161)	309 (164)	295 (157)	303 (161)	306 (162)	302 (161)
Median [Min, Max]	256 [82.4, 780]	256 [82.4, 780]	256 [82.4, 780]	256 [82.4, 780]	256 [82.4, 780]	256 [82.4, 780]

*BMI* Body Mass Index, *SGM* Sexual and Gender Minority, *COPD* Chronic Obstructive Pulmonary Disorder, *HIV* Human Immunodeficiency Virus

13 (0.12%) and 39 (0.36%) respondents refused to respond to the question on HIV testing and HIV risk behaviors, while 360 (3.34%) and 12 (0.11%) individuals responded unknown/unsure to the questions on HIV testing and HIV risk behaviors, respectively. These small values were excluded from the table to protect the confidentiality of these respondents

Most of the respondents were Non-Hispanic White, with 74.8% responding to the HIV testing and HIV risk behavior question. For HIV testing and HIV risk behavior respondents, 88.7% identified as sexual minority individuals alone, 6.8% and 4.7% identified as gender minority individuals alone, and 4.5% and 5.6% identified as both sexual and gender minority individuals, respectively.

### Association of Composite Score of Social Determinants of Health and Sociodemographic Variables with HIV Risk Behaviors

Respondents with high SDOH composite scores (AOR = 1.15, 95% CI = 1.12, 1.19) were more likely to engage in HIV risk behaviors compared with those who had low SDOH composite scores. Respondents who identified as only gender minority individuals (AOR = 0.55, 95% CI = 0.43, 0.69) were less likely to engage in HIV risk behaviors compared to those who identified as sexual minority individuals alone. Respondents who do not have health insurance (AOR = 1.31, 95% CI = 1.06, 1.63) had higher odds of engaging in HIV risk behaviors compared to those who have health insurance. Unemployed respondents (AOR = 0.83, 95% CI = 0.71, 0.97) and respondents who are unable to work (AOR = 0.72, 95% CI = 0.56, 0.93) were less likely to be involved in HIV risk behaviors compared to the employed respondents (Table 2).

**Table 2:** Association of sociodemographic variables and composite score of social determinants of health (SDOH) with HIV testing and HIV risk behaviors

Factors	HIV testing	HIV risk behaviors
Race		
White	Ref	Ref
Non-Hispanic Black	1.955 (1.601,2.386) ***	1.18 (0.946,1.471)
Hispanic	1.218 (1.054,1.408) **	1.191 (1.01,1.405) *
Other	1.019 (0.872,1.19)	1.114 (0.931,1.333)
Age		
18–34	Ref	Ref
35–54	2.413 (2.16,2.695) ***	0.659 (0.581,0.748) ***
55–64	1.43 (1.234,1.658) ***	0.375 (0.306,0.458) ***
65+	0.786 (0.657,0.939) **	0.177 (0.132,0.236) ***
Unknown	1.136 (0.686,1.883)	0.419 (0.194,0.905) *
BMI		
Normal weight	Ref	Ref
Underweight	0.824 (0.616,1.102)	0.929 (0.66,1.307)
Overweight	0.983 (0.894,1.083)	0.917 (0.816,1.03)
Unknown	0.777 (0.629,0.96) *	0.841 (0.635,1.113)
SEX		
Male	Ref	Ref
Female	0.561 (0.513,0.613) ***	0.32 (0.287,0.357) ***
SGM		
SM only	Ref	Ref
GM only	0.485 (0.407,0.578) ***	0.547 (0.427,0.699) ***
SM & GM	0.809 (0.659,0.993) *	0.905 (0.716,1.144)
Education		
< High school	Ref	Ref
>= High school	1.216 (0.975,1.517)	1.17 (0.89,1.538)
Unknown	1.05 (0.31,3.56)	3.061 (0.934,10.028).
Employment Status		
Employed	Ref	Ref
Unemployed	0.665 (0.582,0.761) ***	0.833 (0.712,0.973) *
Retired/homemaker	0.873 (0.744,1.024)	0.882 (0.696,1.119)
Unable to work	1.081 (0.882,1.326)	0.719 (0.559,0.925) *
Unknown	0.868 (0.512,1.471)	1.062 (0.566,1.993)
Income		
< 25k	Ref	Ref
25k − 50k	1.032 (0.887,1.201)	0.95 (0.791,1.141)
50k − 200k	1.047 (0.901,1.217)	0.975 (0.812,1.171)
> 200k	1.219 (0.961,1.547)	1.416 (1.08,1.858) *
Unknown	0.669 (0.566,0.79) ***	0.736 (0.598,0.906) **
Insurance Status		
Yes	Ref	Ref
No	0.836 (0.689,1.013)	1.312 (1.058,1.628) *
Unknown	0.569 (0.451,0.719) ***	0.666 (0.496,0.893) **
Personal Health Care Provider		
Yes	Ref	Ref
No	0.754 (0.666,0.854) ***	0.755 (0.652,0.875) ***
Unknown	0.936 (0.561,1.56)	0.886 (0.481,1.634)
Afford seeing doctors		
Yes	Ref	Ref
No	0.83 (0.726,0.948) **	0.981 (0.844,1.141)
Unknown	0.34 (0.133,0.867) *	0.615 (0.2,1.894)
Asthma History		
No	Ref	Ref
Yes	1.232 (1.11,1.368) ***	1.032 (0.909,1.171)
Unknown	1.019 (0.547,1.901)	0.616 (0.286,1.328)
COPD History		
No	Ref	Ref
Yes	1.522 (1.264,1.833) ***	1.01 (0.799,1.277)
Unknown	1.259 (0.576,2.748)	0.512 (0.172,1.526)
SDOH composite score	1.096 (1.068,1.124) ***	1.151 (1.118,1.185) ***
HIV Prevalence (scaled)	1.122 (1,1.257) *	0.987 (0.896,1.086)

*BMI* Body Mass Index, *SGM* Sexual and Gender Minority, *COPD* Chronic Obstructive Pulmonary Disorder, *HIV* Human Immunodeficiency Virus. * =*P* < 0.05, **=*p* < 0.01, ***=*p* < 0.001

* HIV Prevalence: unit = std = 276 per 100,000 population

### Association of Composite Score of Social Determinants of Health and Sociodemographic Variables with HIV Testing

A positive association was found between the composite score of SDOH (AOR = 1.09, 95% CI = 1.06, 1.12) and HIV testing. Respondents belonging to age groups 35–54 (AOR = 2.41, 95% CI = 2.16, 2.69) and 55–64 (AOR = 1.43, 95% CI = 1.23, 1.66) were more likely to test for HIV compared to those aged 18–34, while older respondents aged 65 and above (AOR = 0.79, 95% CI = 0.66, 0.94) had lesser odds of testing for HIV compared to those between the ages of 18 and 34. Respondents who identified only as gender minority individuals (AOR = 0.49, 95% CI = 0.41, 0.58) were less likely to test for HIV compared to those who only identified as sexual minority individuals (Table 2).

### Association of Sociodemographic Variables and Individual Social Determinants of Health (SDOH) with HIV-Related Risk Behaviors and HIV Testing

Hispanics were (AOR = 1.19, 95% CI = 1.00, 1.40) more likely to engage in HIV risk behaviors and (AOR = 1.22, 95% CI = 1.06, 1.41) more likely to test for HIV compared to Whites, while Non-Hispanic Blacks (AOR = 1.91, 95% CI = 1.56, 2.34) were more likely to test for HIV compared to Whites. Respondents who feel very dissatisfied about life (AOR = 1.20, 95% CI = 1.01, 1.42) were more likely to engage in HIV risk behaviors compared to those who feel satisfied, while unemployed respondents (AOR = 0.652, 95% CI = 0.57, 0.75) were less likely to test for HIV compared to employed respondents (Supplementary Tables 1 and 2).

## Discussion

As one of the first studies examining the relationship between composite SDOH scores and HIV risk behaviors and testing among the SGM population in the US, this research offers key insights into HIV risk and transmission within this group, providing evidence to inform public health interventions aimed at reducing the HIV burden among this vulnerable population.

### Association of Social Determinants of Health and Sociodemographic Variables with HIV Risk Behaviors

Respondents with high SDOH composite scores had higher odds of engaging in HIV risk behaviors compared with those with low SDOH composite scores, providing evidence that the cumulative burden of risk due to SDOH is an important predictor of health, as has been previously demonstrated [20, 21]. The positive association between the composite SDOH score and HIV risk behaviors suggests that individuals facing greater social and structural disadvantages are more likely to engage in behaviors that increase their risk for HIV infection. This implies that addressing the cumulative impact of SDOH, such as poverty, discrimination, and limited access to healthcare, could be critical in reducing HIV risk. Our finding highlights the need for holistic public health interventions that target not just individual behaviors but also the broader social and structural factors that contribute to these risks. Further, it suggests that interventions targeting individuals with higher composite SDOH scores may be particularly beneficial. Feeling dissatisfaction with life was positively associated with increased HIV risk behavior among our respondents, and this behavior could be a maladaptive stress response as reported in previous studies [24, 25].

Gender minority individuals are less likely to engage in HIV risk behavior compared to sexual minority individuals. Internalized stigma and interpersonal stigma have been consistently associated with HIV risk behaviors such as condomless anal sex among gay and bisexual men [26–28], a trend mediated by a lack of routine healthcare utilization [29, 30]. This study also highlighted the relationship between individuals without health insurance and the likelihood of engaging in HIV risk behaviors. A lack of insurance could prevent an individual from utilizing essential healthcare services. Lack of routine health screening and learned distrust of health care services may impact older gay and bisexual men’s willingness to access the available support, education, and counseling to mitigate sexual risk behaviors [29]. Unemployment and being unable to work have negative associations with HIV risk behavior, while having a higher income increases the likelihood of engaging in risky behaviors among our respondents, a counterintuitive relationship that differs from past studies [31, 32]. Although Shi et al. reported that high-income MSM had higher rates of sexual contact with male sex workers and higher rates of payment for sex with men, they found that the rate of condom use was higher among these high-income MSM compared to the low-income group, who were also found to be more involved as male sex workers compared to the high-income earners [32]. The positive association between higher income and HIV risk behavior in our study could mean that individuals with higher income may have a low-risk perception in addition to having the resources to engage in risky activities.

### Association of Social Determinants of Health and Sociodemographic Variables with HIV Testing

Surprisingly, respondents who scored high on the SDOH composite measure have higher odds of testing for HIV compared to those with low SDOH composite scores. Although this appears counterintuitive, individuals with more social disadvantages may be more likely to be exposed to HIV risk behavior, with high-risk perception prompting them to test for HIV. Further, the higher rates of HIV testing among these socially disadvantaged SGM individuals could mean that they have gained increased knowledge about HIV from health promotion programs or services targeted at their neighborhood or social network [33]. Involvement in these social marketing programs may have increased their risk perception toward HIV and potentially increased their access to free or low-cost community clinics and testing sites, hence the higher likelihood of testing for HIV [34, 35]. The feeling of stress was positively associated with HIV testing among our respondents, which differs from past studies [36, 37]. SGM individuals face greater exposure to social and psychological stress due to anti-SGM prejudice, stigma, and discrimination, which can lead to HIV risk behaviors [38, 39]. Minority stress among MSM could predispose them to risky coping behaviors, leading to increased risk perception. This could explain the increased HIV testing rate among our stressed respondents. Further studies need to be conducted among this population to understand this relationship.

The association between HIV testing and age groups in this study varies across different age groups. Older respondents between 35 and 64 years have higher odds of getting tested for HIV compared to younger respondents between 18 and 34 years. This association is consistent with previous studies among Latino and Hispanic MSM [6, 40, 41], which could be accounted for by the increased HIV knowledge among older respondents who may have been exposed to information about HIV testing. However, a negative association was found between HIV testing and older respondents aged 65 and above, which is consistent with past studies [42, 43]. Older MSM may lack the resources and support required to access healthcare services compared to their younger counterparts. Stigma and low-risk perception while in long-term relationships influence HIV testing among older people [44]. These age-related associations with HIV testing suggest the need for tailored HIV testing interventions for younger and older MSM who are at greater risk of HIV infection [45].

Respondents who identified as gender minority individuals alone were less likely to get tested for HIV compared with sexual minority individuals, a finding consistent with a past study [46]. Enacted and internalized stigma has been associated with lower healthcare utilization among LGBTQI + populations [47, 48].

An unexpected association was found between chronic disease and HIV testing in our study, showing that the history of chronic diseases such as asthma and Chronic Obstructive Pulmonary Disease (COPD) increased the odds of HIV testing among the SGM population. Patients who suffer from chronic diseases are likely to have repeated visits to the health facility to monitor their condition [49]. There is a possibility that increased hospital visits improved the HIV testing knowledge among our respondents, which is possible evidence to promote HIV prevention through patient education by healthcare providers. It is also possible that healthcare professionals recommend HIV testing for patients with chronic diseases during their treatment. This area could further be explored among respondents with diverse chronic diseases.

### Strengths and Limitations

The use of large representative data from the BRFSS is a significant strength of this study. BRFSS utilizes validated questions and has a high response rate, which adds to the strength of our study. This is one of the first studies with national data to examine the association between composite SDOH score and HIV risk behavior and HIV testing among SGM. The use of a composite score of SDOH provides evidence that social factors compound to influence health, thus highlighting the need for targeted public health interventions to address multiple social problems simultaneously.

Despite the strengths of this study, some limitations exist. The cross-sectional nature of this study makes it difficult to determine the direction of causation between the variables studied. Longitudinal studies are required to establish a temporal relationship between SDOH and HIV risk behavior and HIV testing among this population. We utilized secondary data for our study, which may have some missing or incomplete data that could potentially introduce some bias in our results.

### Recommendations for Future Research

Reducing the burden of HIV on SGM populations requires a comprehensive approach targeted at dismantling structural factors such as poor access to reliable transportation, unemployment, and lack of health insurance that increase HIV risk behaviors and reduce access to HIV prevention and treatment services among this group. To achieve this, a combination of policy and legal interventions, education, economic support, and community-driven social and political change is needed. The Curriculum of healthcare professionals needs to be revised to enhance diversity, equity, and inclusion training for these professionals. Free or low-cost anonymous HIV testing strategies should be promoted through digital platforms or telehealth. Information about home test kits should be widely disseminated to the public, and the kits should be made accessible with provisions for follow-up care after testing. By addressing the underlying factors that influence individual behavior, rather than the behaviors themselves, these combined efforts can help lower the HIV burden [50].

This study found a significant positive association between the history of chronic disease and HIV testing among MSM. The mechanism of this association is not well understood, thus presenting a critical and understudied area.

## Conclusion

This study demonstrated the combined effect of SDOH in promoting HIV risk behaviors among SGM. Individuals with high SDOH composite scores were found to have higher odds of testing for HIV. This appears counterintuitive, providing an area that could further be explored by future research to understand the mechanism behind this association.

## Supplementary Information

Below is the link to the electronic supplementary material.

Supplementary Material 1
