# Supplementary material for: The Association Between Social Determinants of Health and HIV Risk Behaviors and HIV Testing Among Sexual and Gender Minority Individuals: A National Survey Study
Source: AIDS Behav. 2025 Nov 12;30(4):1030–41. doi: 10.1007/s10461-025-04895-5 (PMC13076379; doi:10.1007/s10461-025-04895-5)
Supplement: Supplementary file 1 — Supplementary Material 1 [file 10461_2025_4895_MOESM1_ESM.docx]

**Supp. Table 1: Association of sociodemographic variables and individual social determinants of health (SDOH) with HIV testing and HIV risk behaviors**

| **Factors** | **HIV testing** | **HIV risk behaviors** |
| --- | --- | --- |
| **Race** |  |  |
| White | Ref | Ref |
| Non-Hispanic Black | 1.911 (1.563,2.338) *** | 1.153 (0.923,1.441) |
| Hispanic | 1.221 (1.055,1.414) ** | 1.186 (1.004,1.4) * |
| Other | 1.009 (0.863,1.181) | 1.111 (0.928,1.331) |
| **Age** |  |  |
| 18-34 | Ref | Ref  **`** |
| 35-54 | 2.404 (2.15,2.69) *** | 0.656 (0.577,0.745) *** |
| 55-64 | 1.476 (1.271,1.714) *** | 0.379 (0.309,0.465) *** |
| 65+ | 0.827 (0.69,0.992) * | 0.181 (0.135,0.242) *** |
| Unknown | 1.193 (0.72,1.975) | 0.431 (0.199,0.933) * |
| **BMI** |  |  |
| Normal weight | Ref | Ref |
| Underweight | 0.847 (0.632,1.135) | 0.942 (0.669,1.326) |
| Over weight | 0.973 (0.884,1.071) | 0.913 (0.812,1.027) |
| Unknown | 0.788 (0.637,0.974) * | 0.842 (0.635,1.115) |
| **SEX** |  |  |
| Male | **Ref** | **Ref** |
| Female | 0.534 (0.488,0.585) *** | 0.311 (0.278,0.348) *** |
| **SGM** |  |  |
| SM only | **Ref** | **Ref** |
| GM only | 0.49 (0.411,0.585) *** | 0.546 (0.427,0.7) *** |
| SM & GM | 0.819 (0.667,1.007). | 0.908 (0.717,1.151) |
| **Education** |  |  |
| < high school | **Ref** | **Ref** |
| >= high school | 1.229 (0.983,1.537) | 1.182 (0.898,1.557) |
| Unknown | 1.068 (0.31,3.68) | 3.076 (0.925,10.224) . |
| **Employment Status** |  |  |
| employed | **Ref** | **Ref** |
| unemployed | 0.652 (0.57,0.747) *** | 0.821 (0.701,0.961) * |
| retired / homemaker | 0.875 (0.745,1.028) | 0.898 (0.707,1.141) |
| unable to work | 1.048 (0.85,1.293) | 0.716 (0.552,0.927) * |
| Unknown | 0.884 (0.521,1.5) | 1.082 (0.576,2.031) |
| **Income** |  |  |
| < 25k | **Ref** | **Ref** |
| 25k - 50k | 1.109 (0.948,1.296) | 0.991 (0.822,1.196) |
| 50k - 200k | 1.149 (0.982,1.344). | 1.04 (0.86,1.258) |
| >200k | 1.346 (1.056,1.715) * | 1.516 (1.15,1.998) ** |
| Unknown | 0.728 (0.613,0.863) *** | 0.771 (0.624,0.953) * |
| **Insurance Status** |  |  |
| Yes | **Ref** | **Ref** |
| No | 0.837 (0.689,1.017) . | 1.296 (1.044,1.61) * |
| Unknown | 0.577 (0.457,0.729) *** | 0.671 (0.5,0.901) ** |
| **Personal Health Care Provider** |  |  |
| Yes | **Ref** | **Ref** |
| No | 0.772 (0.681,0.875) *** | 0.761 (0.657,0.883) *** |
| Unknown | 0.939 (0.56,1.577) | 0.878 (0.474,1.625) |
| **Afford seeing doctors** |  |  |
| Yes | **Ref** | **Ref** |
| No | 0.829 (0.724,0.95) ** | 1.005 (0.862,1.172) |
| Unknown | 0.334 (0.132,0.851) * | 0.589 (0.191,1.818) |
| **Asthma History** |  |  |
| No | **Ref** | **Ref** |
| Yes | 1.219 (1.097,1.354) *** | 1.023 (0.9,1.162) |
| Unknown | 1 (0.536,1.866) | 0.603 (0.279,1.3) |
| **COPD History** |  |  |
| No | **Ref** | **Ref** |
| Yes | 1.527 (1.267,1.842) *** | 0.995 (0.786,1.258) |
| Unknown | 1.274 (0.583,2.785) | 0.535 (0.179,1.596) |
| **HIV_Prevalence (scaled)** | 1.123 (0.999,1.263) | 0.988 (0.898,1.087) |
| * HIV_Prevalence: unit = std = 276 per 100,000 population |  |  |
| **Social Determinants of Health** |  |  |
| **Life Satisfaction** |  |  |
| satisfied | Ref | Ref |
| (very) dissatisfied | 0.957 (0.822,1.114) | 1.2 (1.011,1.423) * |
| **Get Social Support** |  |  |
| always / usually | Ref | Ref |
| sometimes | 0.925 (0.822,1.042) | 1.027 (0.892,1.181) |
| rarely / never | 0.801 (0.676,0.95) | 1.139 (0.936,1.385) |
| **Feel Social Isolation** |  |  |
| rarely / never | Ref | Ref |
| sometimes | 1.01 (0.912,1.119) | 1.003 (0.885,1.138) |
| always / usually | 1.109 (0.955,1.288) | 0.923 (0.773,1.102) |
| **Lost Employment/ Job Reduced** |  |  |
| No | Ref | Ref |
| Yes | 1.236 (1.09,1.4) *** | 1.296 (1.128,1.489) *** |
| **Received Food Stamps** |  |  |
| No | Ref | Ref |
| Yes | 1.466 (1.26,1.705) *** | 1.263 (1.063,1.501) ** |
| **No Money to Buy Food** |  |  |
| rarely / never | Ref | Ref |
| sometimes | 1.013 (0.859,1.195) | 1.19 (0.99,1.431) |
| always / usually | 0.913 (0.737,1.132) | 1.19 (0.938,1.512) |
| **Unable to Pay Mortgage / Rent / Utility** |  |  |
| No | Ref | Ref |
| Yes | 1.285 (1.099,1.504) ** | 1.2 (1.007,1.429) * |
| **Threatened to Shut Off Utility** |  |  |
| No | Ref | Ref |
| Yes | 1.229 (1.03,1.466) * | 1.13 (0.933,1.367) |
| **Lack of Reliable Transportation** |  |  |
| No | Ref | Ref |
| Yes | 1.092 (0.942,1.265) | 1.239 (1.053,1.458) ** |
| **Feel Stress (last 30 days)** |  |  |
| rarely / never | Ref | Ref |
| sometimes | 1.139 (1.023,1.267) * | 1.231 (1.078,1.405) ** |
| always / usually | 1.221 (1.072,1.391) ** | 1.258 (1.077,1.47) ** |

**Supp. Table 2: Association of sociodemographic variables and composite score of social determinants of health (SDOH) with HIV testing and HIV risk behaviors**

| **Factors** | **HIV testing** | **HIV risk behaviors** |
| --- | --- | --- |
| **Race** |  |  |
| White | Ref | Ref |
| Non-Hispanic Black | 1.955 (1.601,2.386) *** | 1.18 (0.946,1.471) |
| Hispanic | 1.218 (1.054,1.408) ** | 1.191 (1.01,1.405) * |
| Other | 1.019 (0.872,1.19) | 1.114 (0.931,1.333) |
| **Age** |  |  |
| 18-34 | Ref | Ref |
| 35-54 | 2.413 (2.16,2.695) *** | 0.659 (0.581,0.748) *** |
| 55-64 | 1.43 (1.234,1.658) *** | 0.375 (0.306,0.458) *** |
| 65+ | 0.786 (0.657,0.939) ** | 0.177 (0.132,0.236) *** |
| Unknown | 1.136 (0.686,1.883) | 0.419 (0.194,0.905) * |
| **BMI** |  |  |
| Normal weight | Ref | Ref |
| Underweight | 0.824 (0.616,1.102) | 0.929 (0.66,1.307) |
| Over weight | 0.983 (0.894,1.083) | 0.917 (0.816,1.03) |
| Unknown | 0.777 (0.629,0.96) * | 0.841 (0.635,1.113) |
| **SEX** |  |  |
| Male | **Ref** | **Ref** |
| Female | 0.561 (0.513,0.613) *** | 0.32 (0.287,0.357) *** |
| **SGM** |  |  |
| SM only | **Ref** | **Ref** |
| GM only | 0.485 (0.407,0.578) *** | 0.547 (0.427,0.699) *** |
| SM & GM | 0.809 (0.659,0.993) * | 0.905 (0.716,1.144) |
| **Education** |  |  |
| < high school | **Ref** | **Ref** |
| >= high school | 1.216 (0.975,1.517) . | 1.17 (0.89,1.538) |
| Unknown | 1.05 (0.31,3.56) | 3.061 (0.934,10.028). |
| **Employment Status** |  |  |
| employed | **Ref** | **Ref** |
| unemployed | 0.665 (0.582,0.761) *** | 0.833 (0.712,0.973) * |
| retired / homemaker | 0.873 (0.744,1.024) | 0.882 (0.696,1.119) |
| unable to work | 1.081 (0.882,1.326) | 0.719 (0.559,0.925) * |
| Unknown | 0.868 (0.512,1.471) | 1.062 (0.566,1.993) |
| **Income** |  |  |
| < 25k | **Ref** | **Ref** |
| 25k - 50k | 1.032 (0.887,1.201) | 0.95 (0.791,1.141) |
| 50k - 200k | 1.047 (0.901,1.217) | 0.975 (0.812,1.171) |
| >200k | 1.219 (0.961,1.547) | 1.416 (1.08,1.858) * |
| Unknown | 0.669 (0.566,0.79) *** | 0.736 (0.598,0.906) ** |
| **Insurance Status** |  |  |
| Yes | **Ref** | **Ref** |
| No | 0.836 (0.689,1.013) | 1.312 (1.058,1.628) * |
| Unknown | 0.569 (0.451,0.719) *** | 0.666 (0.496,0.893) ** |
| **Personal Health Care Provider** |  |  |
| Yes | **Ref** | **Ref** |
| No | 0.754 (0.666,0.854) *** | 0.755 (0.652,0.875) *** |
| Unknown | 0.936 (0.561,1.56) | 0.886 (0.481,1.634) |
| **Afford seeing doctors** |  |  |
| Yes | **Ref** | **Ref** |
| No | 0.83 (0.726,0.948) ** | 0.981 (0.844,1.141) |
| Unknown | 0.34 (0.133,0.867) * | 0.615 (0.2,1.894) |
| **Asthma History** |  |  |
| No | **Ref** | **Ref** |
| Yes | 1.232 (1.11,1.368) *** | 1.032 (0.909,1.171) |
| Unknown | 1.019 (0.547,1.901) | 0.616 (0.286,1.328) |
| **COPD History** |  |  |
| No | **Ref** | **Ref** |
| Yes | 1.522 (1.264,1.833) *** | 1.01 (0.799,1.277) |
| Unknown | 1.259 (0.576,2.748) | 0.512 (0.172,1.526) |
| **SDOH Score** | 1.096 (1.068,1.124) *** | 1.151 (1.118,1.185) *** |
| **HIV_Prevalence (scaled)** | 1.122 (1,1.257) * | 0.987 (0.896,1.086) |
| * HIV_Prevalence: unit = std = 276 per 100,000 population |  |  |

**Supp. Table 3: SDOH/HE Variables with recoding in BRFSS, 2022**

| Variable | 2022 SDOH/HE question with recoding |
| --- | --- |
| Life Satisfaction  (LSATISFY) | In general, how satisfied are you with your life? Are you…  1= Dissatisfied/Very dissatisfied  0=Satisfied/Very satisfied |
| Social and  Emotional Support  (EMTSUPRT) | How often do you get the social and emotional support that you need? Is that…  1= Sometimes/Rarely/Never  0=Always/Usually |
| Social Isolation  (SDHISOLT) | How often do you feel socially isolated from others? Is it…  1= Always/Usually/Sometimes  0=Rarely/Never |
| Employment Stability  (SDHEMPLY) | In the past 12 months, have you lost employment or had hours reduced?  1=Yes  0=No |
| Food Security  (FOODSTMP) | During the past 12 months, have you received food stamps, also called  SNAP, the Supplemental Nutrition Assistance Program, on an EBT card?  1=Yes  0=No |
| Food Security  (SDHFOOD1) | During the past 12 months, how often did the food that you bought not  last, and you didn’t have money to get more? Was that…  1= Always/Usually/Sometimes  0=Rarely/Never |
| Housing Security  (SDHBILLS) | During the last 12 months, was there a time when you were not able to  pay your mortgage, rent, or utility bills?  1=Yes  0=No |
| Utility Security  (SDHUTILS) | During the last 12 months, was there a time when an electric, gas, oil, or  water company threatened to shut off services?  1=Yes  0=No |
| Transportation  Access  (SDHTRNSP) | During the past 12 months, has a lack of reliable transportation kept you  from medical appointments, meetings, work, or getting things  needed for daily living?  1=Yes  0=No |
| Mental Well-being  (SDHSTRE1) | Stress means a situation in which a person feels tense, restless, nervous, or anxious, or is unable to sleep at night because their mind is troubled all the time. Within the last 30 days, how often have you felt this kind of stress? Was it…  1=Always/Usually  0=Sometimes/Rarely/Never |
